# European Health Information Training Programme: a sustainable strategy for strengthening capacity in health information

**DOI:** 10.1093/eurpub/ckae037

**Published:** 2024-07-01

**Authors:** Mariana Peyroteo, Mélanie R Maia, Marília Silva Paulo, Miriam Saso, Nienke Schutte, Petronille Bogaert, Claudia Habl, Luís Velez Lapão, Population Health Information Research Infrastructure

**Affiliations:** UNIDEMI, Department of Mechanical and Industrial Engineering, NOVA School of Science and Technology, Universidade NOVA de Lisboa, Caparica, Portugal; Laboratório Associado de Sistemas Inteligentes, LASI, Guimarães, Portugal; UNIDEMI, Department of Mechanical and Industrial Engineering, NOVA School of Science and Technology, Universidade NOVA de Lisboa, Caparica, Portugal; Laboratório Associado de Sistemas Inteligentes, LASI, Guimarães, Portugal; CHRC, NOVA Medical School, Faculdade de Ciências Médicas, NMS, FCM, Universidade NOVA de Lisboa, Lisboa, Portugal; CHRC, NOVA Medical School, Faculdade de Ciências Médicas, NMS, FCM, Universidade NOVA de Lisboa, Lisboa, Portugal; EU Health Information System Unit, Scientific Directorate of Epidemiology and Public Health, Sciensano, Brussels, Belgium; EU Health Information System Unit, Scientific Directorate of Epidemiology and Public Health, Sciensano, Brussels, Belgium; EU Health Information System Unit, Scientific Directorate of Epidemiology and Public Health, Sciensano, Brussels, Belgium; Gesundheit Österreich GmbH (Austrian National Public Health Institute), Vienna, Austria; UNIDEMI, Department of Mechanical and Industrial Engineering, NOVA School of Science and Technology, Universidade NOVA de Lisboa, Caparica, Portugal; Laboratório Associado de Sistemas Inteligentes, LASI, Guimarães, Portugal; WHO Collaborating Center on Health Workforce Policy and Planning, Instituto de Higiene e Medicina Tropical, Universidade NOVA de Lisboa, Lisboa, Portugal

## Abstract

**Background:**

Before the COVID-19 pandemic, a need for a uniform approach to health information (HI) knowledge in population health analysis across Europe was evident. The Population Health Information Research Infrastructure (PHIRI) emerged as a proactive initiative to strengthen European HI capacities. This article describes the achievements of PHIRI, highlighting its capacity-building activities and their contribution towards a sustainable strategy for the implementation of the European Health Data Space (EHDS).

**Methods:**

PHIRI collaboration established a work package for skill-building activities in population health in partnership with other organizations. Activities included webinars, workshops, sessions, training schools and courses for researchers and public administration workers from Europe and beyond. The primary goal of the activities was to examine the impact of COVID-19 on European health systems at both local and national levels, including healthcare facilities and policymaking entities.

**Results:**

Twelve activities were organized between October 2020 and the summer of 2023. In March 2023, the Spring School on Health Information was organized to share the knowledge achieved from PHIRI and other European Union-related projects. This event also validated the European Health Information Training Programme.

**Conclusions:**

PHIRI’s findings emphasized the importance of equipping the workforce with core HI skills to improve health systems’ preparedness and resilience. Through this research, it is possible to propose a strategy for building capacity that emphasizes the importance of providing training in human-machine dynamics. This approach will contribute to the sustainable implementation of the EHDS.

## Introduction

European Union (EU) countries share the ambition of improving citizens’ health by tackling health inequalities by providing optimal prevention and universal access to a safe, effective and efficient healthcare system i.e. financially and ecologically sustainable.^1^ Timely and high-quality health data and information are needed for evidence generation to support medical decision-making, interventions and health policies, therefore, of imminent importance for improving the population’s health and well-being. Health Information (HI), which includes data on health outcomes, health determinants, health status and health systems performance in Europe, allows for oriented research to increase knowledge and underpin policy decision-making. To make the most of health spending and investments at the EU and national level, health policy and decision-making should be based on robust evidence in the form of high-quality and timely data on population health and health systems’ specific research outcomes.

Since 2015, several European countries have been working to create a sustainable and integrated EU HI research infrastructure to support population health research.

As a result of the ‘BRidging Information and Data Generation for Evidence-based Health Policy and Research’ (BRIDGE Health) Project, the need to create a European Research Infrastructure Consortium to improve the management of HI in the EU that best supports evidence-based health policies and investments became evident.^2^ Following the results of BRIDGE Health, the Joint Action on Health Information (JA *InfAct)* identified the necessity to create a new Distributed Research Infrastructure on Population Health and a comprehensive training program for scientific and administrative staff to improve the availability of comparable, robust and policy-relevant data, and information on health system performance.^3^ Continuing to boost these recommendations, the ‘Population Health Information Research Infrastructure’ (PHIRI) was developed to facilitate and generate the best available evidence for research on the health and well-being of populations impacted by COVID-19.^4^^,^^5^ PHIRI involves national public health institutes, research centres, universities, and ministries of health, fostering collaboration between different perspectives and expertise. Population health data can be crucial in targeted research to increase knowledge and support policymaking based on robust evidence. This robust evidence can only be formed and used if we have the human resources and capacities with the right expertise.

European initiatives such as the European Health Data Space (EHDS),^6^ which aims to facilitate the secure and cross-border exchange of health data within the EU, require specific expertise in both technical and organizational aspects of HI processes. During the COVID-19 pandemic, the shortage of HI experts to tackle the many emerging and demanding ‘health data’ challenges, from specific data collection to more sophisticated data and analysis, almost real-time, has posed some strains on the European member-states’ health systems.

### PHIRI’s capacity-building activities

PHIRI project aims to design a one-stop shop for HI based on four services. One is the provision of capacity-building to ‘promote interoperability and tackle health information inequalities in Europe’, allowing for the cultivation of essential skills that can be effectively applied towards addressing COVID-19 and future crises from a research perspective.^4^ The emergence of new challenges in public health, such as pandemics, climate change or digital transformation, calls for highly qualified health experts who can respond promptly and effectively. However, despite the growing need for skilled public health professionals, there are significant inequalities in the availability of and training opportunities for such individuals across Europe. Some countries may have well-established systems and sufficient workforce, while others may lack the necessary infrastructure and expertise. This disparity can hinder the effective management of HI and impede the implementation of evidence-based strategies to address these public health challenges. To bridge these inequalities, investing in capacity-building, collaborative initiatives, and training programs that focus on developing a qualified and competent workforce in public HI and advancing health data analytics.

PHIRI provides capacity-building actions in HI and strengthens capacity at the European level on innovative research methods on population health developed during the COVID-19 pandemic, e.g. HI system assessments, creating metadata records, infodemics and federated analyses of research queries. As such, this article aims to present an observational description of the comprehensive work developed within the PHIRI capacity-building work package to strengthen the public health workforce’s HI knowledge at the European level and reduce inequalities for COVID-19 and future pandemics.

## Methods

Based on the conceptual model of the PHIRI project, the activities were carried out within the research topics addressed throughout data to policy. The activities were based on a pedagogical approach, complementing the sharing of theoretical knowledge with practical and real-world cases. The use of practical, engaging methodologies was promoted, allowing participants to share their doubts and questions with all the speakers.

All work was developed by different partners and promoted collaboration with external teams. This approach provides national population health experts with tailored knowledge i.e. important to respond to the different needs based on their training and healthcare systems.

Due to pandemic restrictions, most PHIRI project activities were conducted remotely, including webinars, workshops, training schools and courses. These activities were aimed at national public health institutes, universities and decision-makers in population health. The details of these activities can be found under the tab ‘PHIRI activities’ in the ‘search training’ section of the European Health Information Portal (HIP).^7^

In the results section, the activities were analyzed based on the topic, format and the countries and institutions of the participating partners to highlight the importance of collaborations between European institutions.

## Results

Twelve capacity-building activities were organized based on the consortium’s expertise and the identified needs.^8–22^ A summary of each activity is presented in table 1.

**Table 1:** Description of capacity-building activities organized in the Population Health Information Research Infrastructure Project

Title	Date, time and format	Description	Topic	Organizing partners	Ref.
‘Digital and Innovative Tools: the challenges of contact tracing in Public Health’	15 February 2022 10:30–12:00 h Online	This webinar featured a comprehensive agenda covering various aspects of contact tracing. It included an introduction to PHIRI/WP5 context, the role of digital and innovative tools in public health and COVID-19, a perspective on contact tracing, an overview of contact tracing in Europe, contact tracing modelling, a discussion, and a concluding wrap-up and closing session. These sessions collectively explored the multifaceted challenges and innovations within contact tracing in public health.	Contact Tracing Response Plan Medical Technology COVID-19	Germany (RKI) Portugal (UNL)	
‘Fact-checking COVID-19: joint UnCoVer & PHIRI workshop’	30 June 2021 14:00–16:00 h Online	During the workshop, after introducing the UnCoVer and PHIRI projects, a diverse range of experts, including those with backgrounds in big data and journalism, will discuss the vital yet challenging task of effectively communicating science to the general public. Following these presentations, experts from the University of Liverpool and the University of Dundee will introduce participants to the FakeNews Immunity chatbot, an incredible tool designed to enhance individuals’ resilience to misinformation and disinformation by teaching them how to identify fallacies and recognize misleading or deceptive stories. The session will culminate in breakout rooms, where participants can explore the tool and gain insights into fallacies from renowned philosophers such as Aristotle, Gorgias, and Socrates.	Infodemic	Belgium (Sciensano) Norway (NIPH) Portugal (UNL) Spain (ISCIII) United Kingdom (UnCoVer)	
‘Burden of disease assessment for COVID-19: initial insights and future perspectives’	21 May 2021 14:00–15:00 h Online	In the midst of the COVID-19 pandemic, several countries are actively assessing the health consequences it has on their population. The burden of disease framework provides a means to conduct thorough and standardized assessments of the pandemic’s impact on population health, enabling comparisons with other diseases and risk factors. This webinar presented initial estimates of the population-level burden of COVID-19 and focused on post-COVID conditions, which represent a substantial yet uncertain component of the overall COVID-19 disease burden.	Burden of disease	Belgium (ITM) Germany (RKI) United Kingdom (PH Scotland) United States of America (NIAID)	
‘Medicinal therapies for COVID-19 and its impact on population health’	25 January 2022 10:30–12:00 h Online	This webinar comprises an introduction, a presentation on COVID-19 medicines, a perspective from payers, a discussion on the impact of medicinal therapies on COVID-19 vaccination, and a moderated discussion.	Vaccination Coverage COVID-19 Medicinal Therapies	Austria (EUNetHTA, GÖG) Belgium (Sciensano, RIZIV/INAMI) Brazil (University of São Paulo)	
‘COVID-19 Burden of disease training school’	30 March –1 April 2022 In person	The Training School (TS) introduces burden of disease assessment, using COVID-19 as a case study. The TS will provide public health professionals, and researchers from related fields, with practical knowledge about summary measures of population health, the historical background of the Global Burden of Disease study and its outputs. Furthermore, the TS will introduce the concept and rationale of the main metrics (Years of life lost, years lived with disability and Disability-Adjusted Life Years), and underlines their application and importance or priority setting in public health policy and decision-making processes.	COVID-19 Burden of disease	Belgium (Sciensano) EU-COST Action on Burden of disease Denmark (NFI-DTU) The Netherlands (RIVM)	
‘Monitoring COVID-19 related changes in mental health in Europe’	19 May 2022 12:30–13:30 h Online	In the framework of PHIRI, different European countries are conducting research through a used case on the COVID-19 related changes in population mental health analysing register data. This webinar, addressed to a wide audience of researchers, stakeholders and decision makers, will present the rationale, implementation process and results.	COVID-19 Mental diseases	Austria (GÖG) Germany (RKI) Spain (ISCIII) United Kingdom (Swansea University)	
‘Toward foresight informed policy’	19 September 2022 09:00–11:30 h Online	The workshop aimed to enhance understanding of past national policies related to Public Health Foresight with the goal of improving both direct and indirect health impacts, including those related to COVID-19. The workshop also sought to identify examples of successful and less successful foresight practices across Europe. The event featured a keynote introduction to the topic, a showcase of PHIRI Foresighting activities, and a moderated roundtable debate involving decision makers and recipients of forecasting from five European countries, overseeing the session. The workshop provided key insights into the field of public health foresight and its impact on past policy and health outcomes.	COVID-19 Policy Foresight Scenarios	Austria (GÖG) European Parliament Hungary (NNK) Malta (MfH) Portugal (UNL) Spain (ISCIII) The Netherlands (RIVM)	
‘European experiences, lessons learned and good practices on infodemic management during the COVID-19 pandemic’	9 June 2021 09:00–13:30 h 16 June 2021 09:00–13:30 h Online	This workshop aimed to gather and present experiences, lessons learned, and good practices related to infodemic management during the COVID-19 pandemic in Europe. Infodemic management had been approached in various ways in European countries, contingent on their capacities, available resources, and the involved institutions. The event sought to address questions such as the strategies that were attempted, their effectiveness, challenges encountered, the roles of different professionals in infodemic management, and the usefulness of specific tools. The workshop featured speakers from diverse European countries with varied backgrounds and experiences, who shared their national infodemic management practices and engaged in discussions with each other and the audience. Additionally, practical tools and exercises for infodemic management were proposed during the workshop.	Infodemic	Belgium (Sciensano) ECDC European Science Media Hub Finland (THL) France (Institut Pasteur) Germany (WiD) Italy (Pagella Politica) Poland (PPSF) Romania (INSP) Serbia (UBFM) Spain (ISCIII) Switzerland (UNILU) World Health Organization	
‘Learning from journeys in literature reviews of COVID-19 research’	11 October 2022 10:30–12:30 h Online	In the capacity-building webinar, the PHIRI project members shared their experiences in applying various approaches to manage literature reviews summarizing COVID-19 research. The course began with a presentation that explained the different types of reviews and covered fundamental concepts related to literature reviews in population studies, including methodological steps and outcome distinctions. Following this, researchers from PHIRI shared their experiences in conducting literature reviews focused on COVID-19 evidence. The session concluded with a practical exercise that involved using the free online tool Rayyan, providing participants with hands-on experience in this vital research methodology.	Research Literature Review Qualitative Research	France (SPF) Portugal (UNL, FMUL) Spain (ISCIII)	
‘PHIRI Foresight capacity building course’	25 March–28 October 2021 Online	The Foresight Capacity Building course aimed to develop and provide foresight capacity for all European Member States. Its primary goal was to standardize the knowledge required for performing foresight, bridging information gaps, and fostering uniformity in European data, while simultaneously fostering collaboration among Member States in the domain of foresight studies. The course consisted of five modules. The first module introduced the basics of conducting foresight studies, covering three core aspects: Purpose & Methodology (‘why’ and ‘how’), Process & Participation (‘how’ and ‘with whom’), and Product and Communication (‘what’ and ‘for whom’). The second module explored the value of foresight studies, their aims, objectives, and the importance of various stakeholders. Module three provided insights into data identification, methodologies, analysis, and result interpretation for foresight studies. Module four focused on disseminating results effectively to stakeholders and policymakers. Lastly, the fifth module synthesized lessons, evaluated the course, and addressed participants’ questions regarding planning and conducting their own foresight studies.	Policy Foresight Scenarios	Austria (GÖG) Belgium (Sciensano) EuroHealthNet France (AMSE) Portugal (UNL) The Netherlands (RIVM)	
‘A tour through Europe: mapping health information systems for COVID-19 and a future EHDS’	23 June 2022 10:00–12:00 h Online	This webinar showcased the initial findings of the PHIRI COVID-19 Health Information System assessments and provided updates on the TEHDAS country visits, focusing on the mapping of health data management systems and readiness to integrate into the European Health Data Space (EHDS). The presentation offered valuable insights into these assessments, conducted in five European countries for COVID-19 Health Information Systems, and six countries with respect to their preparedness for EHDS participation.	COVID-19 Policy Health System Performance Health Information System	Belgium (Sciensano)	
‘Spring School on Health Information: COVID-19 Health Information Innovations for the Future’	2 March–28 March 2023 Online	The 2023 edition of the SSHI covered various topics, including innovative health information data collection, sources, metrics, and indicators, health data analysis and interpretation, the transition from health data to policy, interoperability, and clinical practice, data protection, and ethical considerations in health information, as well as strategies for ensuring a sustainable future for health information in Europe to better prepare for future pandemics. The program commenced with a keynote introduction to the theme and a brief showcase of PHIRI’s Health Information activities, followed by presentations highlighting COVID-19 health information innovations across European countries, allowing for questions and discussions after each session. Additionally, participants who attended 80% of the sessions (4 out of 5) and completed all exercises, including a final essay on COVID-19 innovation, were eligible to receive a certificate of achievement.	Communication COVID-19 Policy Health Systems Innovation Health Information	Austria (GÖG) Belgium (Sciensano) ECDC Finland (THL) Greece (UOC) Hungary (OKFO) Italy (ISS) Malta (MfH) Portugal (UNL, DGS, INESC-TEC) Spain (IACS) The Netherlands (RIVM) Turkey (Hacettepe University) World Health Organization	

Note: AMSE, Aix-Marseille School of Economics; BRIDGE Health, BRidging Information and Data Generation for Evidence-based Health Policy and Research Project; DGS, general directorate of health; ECDC, European Centre for Disease Prevention and Control; EHMA, European Health Management Association; EPHC, European Public Health Conference; EUNetHTA, European Network for Health Technology Assessment; FMUL, Faculdade de Medicina da Universidade de Lisboa; GÖG, Gesundheit Österreich GmbH; IACS, Instituto Aragonés de Ciencias de la Salud; INESC-TEC, Instituto de Engenharia de Sistemas e Computadores, Tecnologia e Ciência; INSP, Institutul Național de Sănătate Publică; ISCIII, Instituto De Salud Carlos III; ISS, Istituto Superiore di Sanità; ITM, Institute of Tropical Medicine, Universiteit Antwerpen; JA InfAct, Joint Action on Health Information; MfH, Ministry for Health, Malta; NFI-DTU, National Food Institute-Technical University of Denmark; NIAID, National Institute of Allergy and Infectious Diseases; NIPH, Norwegian Institute of Public Health; NNK, Nemzeti Népegészségügyi Központ; OKFO, Országos Kórházi Főigazgatóság; PHIRI, Population Health Information Research Infrastructure; PPSF, Poland Patients Safety Foundation; RIVM, Rijksinstituut voor Volksgezondheid en Milieu; RIZIV/INAMI, Rijksinstituut voor Ziekte- en Invaliditeitsverzekering; RKI, Robert Koch-Institut; SPF, Santé publique France; THL, Terveyden ja hyvinvoinnin laitos; UBFM, University of Belgrade—Faculty of Medicine; UNILU, University of Luxembourg; UNL, Universidade NOVA de Lisboa; UOC, University of Crete; WiD, Wissenschaft im Dialog.

Half of the activities were in the format of webinars (50%), followed by workshops (25%) and courses (25%). The topics focused varied from focusing on COVID-19 (58%), policies (33,3%), infodemic (17%) and burden of disease (17%). These initiatives involved 41 partners from 17 countries, four international organizations, and three project collaborations.

PHIRI has also provided over 60 workshops at conferences such as the European Public Health Conference, The European Health Management Association annual meeting, the World Congress on Public Health and during many of the European Public Health Weeks every year in May.

### Spring School on Health Information

Following the lessons learned from the 1st European School of Health Information of the JA InfAct,^23^ a so-called Spring School on Health Information (SSHI) was proposed to provide cost-free practical knowledge on both European and national approaches to HI, focusing on the innovative tools developed during the COVID-19 pandemic. Based on Zachman’s information architecture framework,^24^ the course was organized into five online sessions, and each day was dedicated to a relevant topic covering a range of aspects from Data Collection to Policy Dialogue and Knowledge Translation. The pedagogical approach integrated a theoretical (lectures from experts in the field) and practical (home assignment and class discussions) component. The curriculum was developed as a comprehensive training resource for public health professionals, serving as a proof of concept that can be utilized in current and future national, European and international training activities.

The 2023 edition of the SSHI counted 27 lecturers from 15 distinct nations, led by reference experts in their HI systems field. In total, 49 persons (public health professionals, health data scientists, communication experts, young researchers and policy-makers) from 26 nations (European and non-European countries, such as Asia and Africa) attended the seminar series for 20 h. From these, 24 concluded the school with a certificate as they had delivered the required final essays.

### European Health Information Training Programme

HI is a comprehensive area in a maturing process. It includes data collection, data analysis and inference, indicators development, data presentation, ethics, data protection, infodemics, information management and translational research for developing new policies. As a result of previous research on the JA InfAct, it was clear that knowledge and capacities on HI vary among European countries and that there is a need to improve common mechanisms for strengthening the capacity to use and manage HI.^25^ As such, a European Health Information Training Programme (EHITP) was established and led to the developing of a Roadmap for the Sustainability of Health Information in Europe.^26^

Being capacity-building one of the pillars of PHIRI, it was possible to achieve the following contributions:

- **Concepts**: The activities started with an introduction that covered a range of topics in public health, such as infodemic, mental health and burden of disease, and digital health, including contact tracing and HI systems.
- **Research**: In addition to the concepts, training activities were carried out to foster research skills, such as using foresight methodologies and systematic literature reviews.
- **Capacity-building**: Building on the 1st European School of Health Information (2020) and adding the results of the work throughout PHIRI, the SSHI was the validation of the concept of EHITP, to be organized every spring from now on. The edition of 2024 is already being planned to focus on HI preparedness and resilience.
- **European strategy**: The activities were designed for a diverse range of targets, enabling participants to apply the knowledge they gained to their unique contexts, making the EHDS strategy a central inspiration.
- **Spring School on Health Information as a European flagship training on health Information**: SSHI reflected on the innovations achieved during the COVID-19 pandemic, considering the processes of collecting metrics and indicators, interpretation and analysis of data, knowledge transfer and interoperability of information; ethical issues and the General Data Protection Regulation (GDPR) related to HI; foresight, preparedness and sustainability for future crises.
- **Collaboration**: Various entities supported PHIRI’s capacity-building to increase their effectiveness. These included international organizations such as the WHO and European Centre for Disease Prevention and Control (ECDC), EU-funded projects such as TEHDAS^27^ and UnCoVer,^28^ and the collaboration of different EU countries involved in the project.
- **Learning & sharing**: The activities under PHIRI are designed to create a network of experts and researchers focused on improving population health through capacity-building. In addition to capacity-building activities, 66 activities were organized to share the knowledge and results gained during the project at European conferences.
- **Vision**: PHIRI capacity-building activities are designed to enhance competencies in the sharing of secondary data.
- **Milestones**: As reported by the InfAct,^29^ there was a need to create a single place to access the training activities carried out in HI. The establishment of the European HIP, which includes a capacity-building section, was successfully achieved through the PHIRI Project.
- **Sustainability**: To ensure sustainability, PHIRI’s EU member state partners are committed to keeping the HIP current and operational, like the new SSHI edition in 2024. In light of the positive outcomes, collaborations are underway with international entities such as the ECDC Virtual Academy, OECD and WHO, and an informal network established within PHIRI.

## Discussion

Robust and efficient HI systems are vital for evidence-informed decision-making. They provide the necessary infrastructure to collect, manage and analyze health data, enabling policymakers and health professionals to make informed choices and implement effective interventions. Especially in times of crisis, it is essential to have a skilled and knowledgeable workforce capable of supporting the functioning of HI Systems. However, previous research and the COVID-19 pandemic highlighted disparities in the availability and training of qualified HI professionals across Europe, posing significant challenges to countries’ ability to respond effectively to health challenges.

Based on the last three years of work, there are three stages to address public health scenarios that reflect the need for more qualified Public Health Information professionals and capacity-building activities:

- The *status quo*, where the current pattern (collective intelligence) will continue to be followed, bridging the existing inequities between different countries about essential population HI skills^30^;
- The evolution of an emergent and external phenomenon that challenges the resilience of healthcare systems highlights the need to empower the public health workforce and health data experts for sustainability, preparedness and the ability to innovate health systems in times of crisis.
- To establish EHDS, the evolution in human-machine intimacy (e.g. explainable artificial intelligence) and collaboration will be evident. There is a need to provide training and skills on how to integrate information systems and technologies in real architectures of real human and machine learning dynamics, where humans will need to set up the framework for machine-to-machine interaction.

Joint efforts are needed to develop and implement initiatives that support and provide learning opportunities. The high participation and interest in PHIRI’s capacity-building activities show the recognition among health professionals of training’s crucial role in personal development and practical HI management. These activities equip professionals with the knowledge and skills to adapt to the ever-changing health landscape and overcome new challenges.

**Figure 1:**
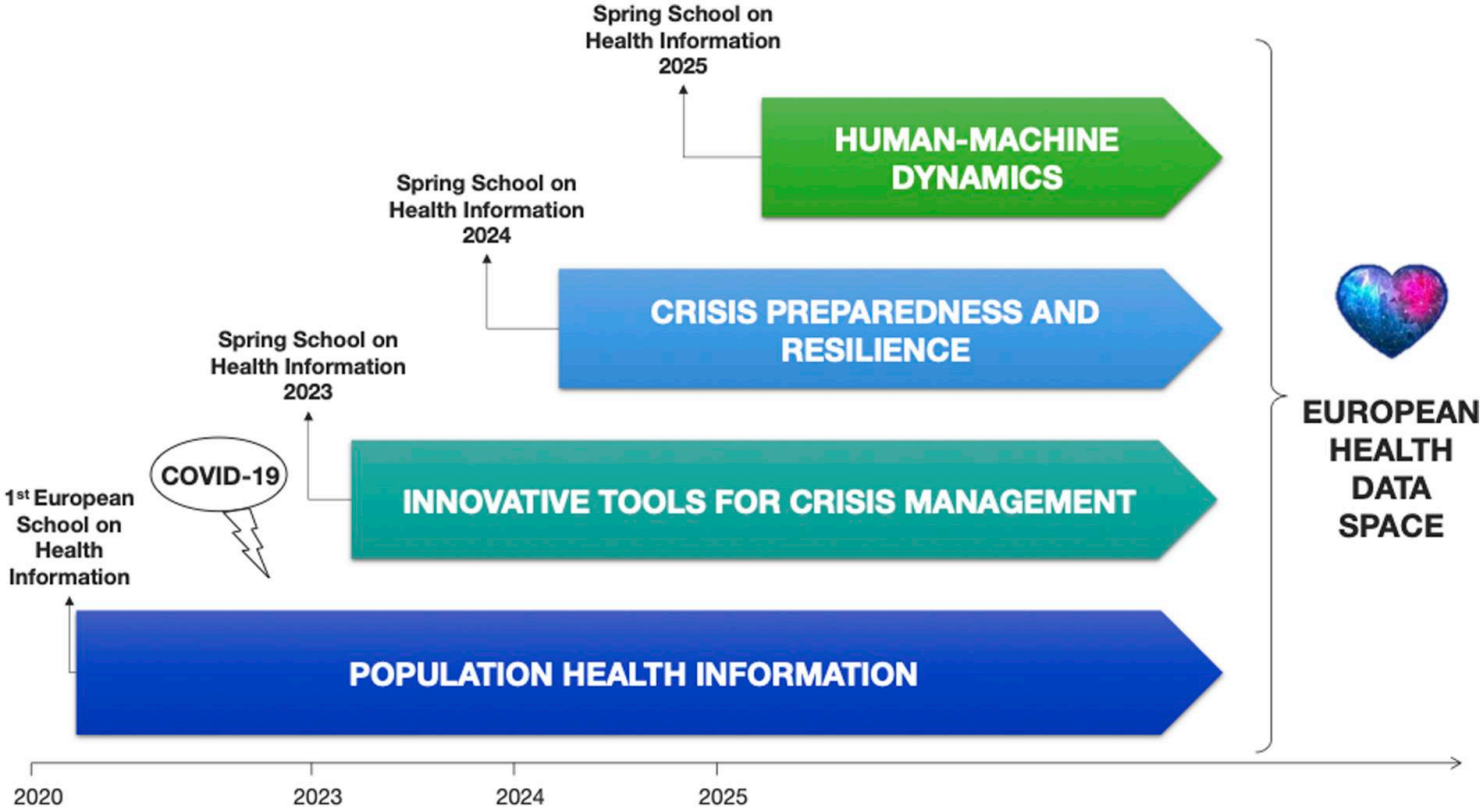
Capacity-building strategy for sustainable implementation of the European Health Data Space

Collaboration within PHIRI allowed an exchange of empirical experiences, translating them into a robust, validated roadmap. This roadmap outlines a strategic direction for the evolving healthcare landscape. It addresses what we must do and what has to be in the future, as healthcare and healthcare-related professionals in this landscape continuously change in healthcare systems locally and globally. Figure 1 illustrates the evolution of these stages, proposing a strategy for achieving consistent and sustainable EHDS implementation across Europe until 2025. It emphasizes the importance of the EHITP and the acquisition of essential human-machine dynamic skills (e.g. artificial intelligence, federated analysis data infrastructures, GDPR, among others), paying attention to critical skills in population HI and crisis preparedness and resilience skills.

## Conclusions

The PHIRI project builds upon the EHITP and effectively addresses knowledge gaps in health informatics, emphasizing the importance of sustainability in implementing electronic health data systems. This commitment requires following the EHITP, including an annual SSHI, in line with its strategic vision.

Apart from having a clear vision, ensuring and maintaining collaboration is crucial. The PHIRI team's dedication to continue promoting capacity-building activities in health informatics, in partnership with OECD, ECDC and WHO, is a significant effort to enhance health informatics across EU member states and reduce disparities. Collaboration among several European universities and public health institutes is crucial in strengthening Europe's preparedness and resilience against future pandemics. This consortium also aims to ensure a consistent and sustainable transition to the EHDS while embracing digital advancements.

## Data Availability

The data underlying this article are available at the European Health Information Portal at https://www.healthinformationportal.eu/activities/catalogue.
Key points Cross-country exchange and mutual learning are impetus tools to leverage the HI knowledge and expertise that exists and is available to contribute to the improvement of Europe’s resilience to the health crisis. The PHIRI capacity-building activities strategy follows the Sustainability Roadmap introduced by JA InfAct, ensuring consistency. A need has been identified for courses focusing on different stakeholders, from more specific topics (infodemic management, metadata) to more comprehensive training (foresight, SSHI). The strategy developed emphasizes collaboration, where European projects have contributed to mitigating European health information inequalities. New digital tools for population health are emerging, requiring adequate multidisciplinary training, and the European context is an excellent lever to bring in the best experts on the topics.
